# Polymorphism in merozoite surface protein-7E of *Plasmodium vivax* in Thailand: Natural selection related to protein secondary structure

**DOI:** 10.1371/journal.pone.0196765

**Published:** 2018-05-02

**Authors:** Chew Weng Cheng, Chaturong Putaporntip, Somchai Jongwutiwes

**Affiliations:** Molecular Biology of Malaria and Opportunistic Parasites Research Unit, Department of Parasitology, Faculty of Medicine, Chulalongkorn University, Bangkok, Thailand; Universidade Federal de Minas Gerais, BRAZIL

## Abstract

Merozoite surface protein 7 (MSP-7) is a multigene family expressed during malaria blood-stage infection. MSP-7 forms complex with MSP-1 prior to merozoite egress from erythrocytes, and could affect merozoite invasion of erythrocytes. To characterize sequence variation in the orthologue in *P*. *vivax* (PvMSP-7), a gene member encoding PvMSP-7E was analyzed among 92 Thai isolates collected from 3 major endemic areas of Thailand (Northwest: Tak, Northeast: Ubon Ratchathani, and South: Yala and Narathiwat provinces). In total, 52 distinct haplotypes were found to circulate in these areas. Although population structure based on this locus was observed between each endemic area, no genetic differentiation occurred between populations collected from different periods in the same endemic area, suggesting spatial but not temporal genetic variation. Sequence microheterogeneity in both N- and C- terminal regions was predicted to display 4 and 6 α-helical domains, respectively. Signals of purifying selection were observed in α-helices II-X, suggesting structural or functional constraint in these domains. By contrast, α-helix-I spanning the putative signal peptide was under positive selection, in which amino acid substitutions could alter predicted CD4+ T helper cell epitopes. The central region of *PvMSP-7E* comprised the 5’-trimorphic and the 3’-dimorphic subregions. Positive selection was identified in the 3’ dimorphic subregion of the central domain. A consensus of intrinsically unstructured or disordered protein was predicted to encompass the entire central domain that contained a number of putative B cell epitopes and putative protein binding regions. Evidences of intragenic recombination were more common in the central region than the remainders of the gene. These results suggest that the extent of sequence variation, recombination events and selective pressures in the *PvMSP-7E* locus seem to be differentially affected by protein secondary structure.

## Introduction

In most malaria endemic areas outside of Africa, *Plasmodium vivax* mainly coexists with *Plasmodium falciparum*, both of which affect global health burden and contribute remarkably to economic loss [1, 2]. Control of malaria caused by *P*. *vivax* has been complicated by the presence of hypnozoites responsible for chronic relapsing illness, the emergence of chloroquine-resistant *P*. *vivax* strains and spread of insecticide-resistant anopheline vectors [3]. Therefore, alternative measures are required such as development of malaria vaccines [4].

One of the prime strategies for asexual blood stage vaccine development is to mount immunity that interrupts the invasion of *Plasmodium* merozoites into erythrocytes [5]. The initial attachment of merozoite to erythrocyte surface is primarily mediated by the binding of merozoite surface protein-1 (MSP-1) to Band 3 on the erythrocyte membrane [6]. Although MSP-1 has been considered a prime target for asexual blood stage vaccine development, recent studies have shown that other merozoite surface proteins, such as merozoite surface proteins-6 and -7 (MSP-6 and MSP-7), form a non-covalent complex with MSP-1 prior to receptor-ligand recognition [7–10]. MSP-7 is expressed during schizogony and undergoes two steps of proteolytic processing akin to MSP-1. Disruption of *P*. *falciparum* MSP-7 (PfMSP-7) has resulted in partial impairment in erythrocyte invasion by malarial merozoites [11]. Meanwhile, anti-PfMSP-1/6/7 antibodies can interfere with MSP-1 shedding and reduce merozoite invasion into erythrocytes [12]. Disruption of the orthologous gene in *P*. *berghei* affected intraerythrocytic growth of parasites [13]. Furthermore, specific binding of *P*. *berghei* MSP-7 to P-selectin has suggested the role of this protein in modulating disease severity through immunological process [14]. Therefore, immunity induced by vaccines derived from malarial MSP-7 could potentially interrupt parasite development.

MSP-7 proteins are encoded by a multigene family, of which the number of gene members varies across *Plasmodium* species [15, 16]. The *MSP7* family of *P*. *vivax* contains 13 gene members, designated alphabetically from *PvMSP-7A* to *PvMSP-7M*, found in tandem with head to tail arrangement on chromosome 12 [15]. Of these, *PvMSP-7C*, *-7E*, *-7H* and *-7I* displayed higher nucleotide diversity than other paralogous gene members [17–19]. Although the less polymorphic protein members have been suggested for vaccine incorporation, it is currently unknown whether particular members of the PvMSP7 family involve in binding to PvMSP1 during host cell invasion. Importantly, several malarial surface proteins involved in host cell invasion are under positive or balancing selection and have been considered to be targets of vaccine development [20]. Meanwhile, antigenic diversity in several malarial vaccine candidates could elicit allele-specific immune responses, an issue that may complicate vaccine design. Therefore, analysis of sequence variation in malarial vaccine candidates among parasite populations from different geographic areas is essential for a basis of vaccine development [4].

The extent of genetic diversity in *PvMSP-7* has been analyzed by using *P*. *vivax* samples from Colombia [17–19]. However, PvMSP7E seems to be the most polymorphic protein member and reportedly has evolved rather rapidly [19]. Therefore, this locus could be useful as a marker for *P*. *vivax* populations besides being a member in the protein family involved in erythrocyte invasion. The aim of this study is to analyze sequence diversity at this locus among *P*. *vivax* populations from diverse malaria endemic areas of Thailand. Results revealed extensive diversity in *PvMSP7E* of Thai isolates that exhibited spatial variation. Furthermore, differential selective pressures in the *PvMSP-7E* locus seem to be related with its predicted protein secondary structure.

## Materials and methods

### Human ethics statement

Written informed consent was obtained from all participants or from their parents or guardians prior to blood sample collection. Research protocol was approved by the Institutional Review Board in Human Research of Faculty of Medicine, Chulalongkorn University, Thailand (IRB No. 104/59).

### Study population

One hundred and ten venous blood samples were collected from uncomplicated symptomatic *P*. *vivax*-infected patients diagnosed by microscopic examination of Giemsa stained blood films and preserved in EDTA anticoagulant. Of these, 80 samples were collected from Tak (n = 31), Narathiwat (n = 16) and Yala (n = 9) provinces during 2008–2009. Additional 24 blood samples from Tak province preserved at -80°C were obtained in 1996. Thirty samples from Ubon Ratchathani province was collected during a malaria epidemic in 2014–2015. All blood samples were stored at -30°C.

### DNA extraction

Genomic DNA of blood samples was prepared by using QIAamp DNA mini kit (Qiagen, Hilden, Germany) essentially per manufacturer’s recommendations. DNA was stored at -30 °C until use.

### PCR detection of *P*. *vivax* and genotyping

All blood samples were reaffirmed for the presence of *P*. *vivax* DNA by nested PCR method using specific primers derived from the *18S rRNA* gene as described previously [21]. Allele-specific PCR targeting the polymorphic block 6 of the merozoite surface protein 1 of *P*. *vivax* was deployed to determine the number of parasite clones in each isolate as previously reported [22–24].

### Amplification and sequencing of *PvMSP-7E*

The complete coding region of *PvMSP-7E* (~1.1 kb) from each isolate was amplified by nested PCR using outer primers PvMSP-7F (5’-CATACCTTCGATACGTGTACTTC-3’) and PvMSP-7R (5’-CATTTCGCGTGTGCGTGTCTATG-3’) of the Salvador I strain (GenBank accession no. XM_001614084, chromosome 12: position 771164 to 772282) and the inner primers located 5’ and 3’ before and after the coding region of PvMSP-7E (PvMSP-7EF: 5’-AATCGCCACACATCGTCTGTG-3’ and PvMSP-7ER: 5’-ATTTCATCTTTACTGTTGGGCAC-3’) (S1 Fig). Primary PCR amplification was performed in a total volume of 15 μL containing PCR buffer, 200 μM dNTP, 0.2 μM of each primer, nuclease free water, 2 μL of template DNA and 1.25 units of TaKaRa LA Taq^™^ (Takara, Seta, Japan). The thermal cycling profiles for primary PCR contained a pre-amplification denaturation at 94°C, 60 s; followed by 35 cycles of denaturation at 96°C, 30 s; annealing at 50°C, 30 s; polymerization at 72°C, 7 minutes, and final elongation at 72°C, 10 minutes. Secondary PCR contained PCR buffer, 200 μM dNTP, 0.2 μM of each primer, nuclease free water, 1 μL of template DNA from primary PCR and 1.25 units of ExTaq DNA polymerase (Takara, Seta, Japan) in a total volume of 30 μL. The amplification profile for secondary PCR consisted of 94°C, 60 s; 30 cycles of 96°C, 30 s; 50°C, 30 s and 72°C, 2 minutes; and 72°C, 5 minutes. DNA amplification was performed by using a GeneAmp 9700 PCR thermal cycler (Applied Biosystems, Foster City, CA). The PCR products were analyzed on 1% agarose gel electrophoresis, stained with ethidium bromide and visualized under UV transillumination. Template DNA for sequencing was prepared from PCR products that were purified by using QIAquick PCR purification kit (Qiagen, Hilden, Germany). DNA sequences were determined directly and bi-directionally from the purified templates from secondary PCR using ABI PRISM BigDye Terminator v3.1 Ready Reaction Cycle Sequencing kit (Applied Biosystems) and sequencing primers.

### Data analysis

DNA sequences were aligned using the default option in CLUSTAL W program [25]. The *PvMSP-7E* gene of the Salvador I strain was used as a reference sequence (PVX_082665). Sequences from the Colombian isolates previously reported were also included for comparison (GenBank accession nos. KM212276-KM212294) [19]. All sites at which the alignment postulated a gap were eliminated in pairwise comparisons of the analysis. Exploration of repetitive DNA sequence motifs was performed by scanning the sequence with Tandem Repeats Finder version 4.0 program [26]. Protein secondary structure prediction was determined by Deep Convolutional Neural Filed program (DeepCNF) implemented in the RaptorX-Property Web-Server [27]. The DeepCNF method has been validated to outperform other methods that achieved >70% accuracy in eight-state protein structure prediction [28]. Protein disordered or intrinsically unstructured regions were analyzed by using the GeneSilico MetaDisorder service [29]. Prediction of anchorage or protein-protein interaction regions in disordered proteins was done by using the ANCHOR/IUPRED web server [30]. Haplotype diversity and its sampling variance were calculated using the DnaSP version 5.10 program [31]. Nucleotide diversity (π) was calculated from the mean of pairwise sequence differences in the sample sequences using Juke and Cantor model of nucleotide substitution [32]. The number of synonymous substitutions per synonymous site (*d*_*S*_) and the number of nonsynonymous substitutions per nonsynonymous site (*d*_*N*_) were computed by Nei and Gojobori’s model with Jukes-Cantor correction [32, 33]. The standard errors of these parameters were estimated by the bootstrap method with 1,000 pseudosamplings implemented in the MEGA 6.0 program [34]. Statistical differences between these parameters were determined by a two-tailed Z-test and the significance level was set at *p* < 0.05. Codon-based analyses of selection were performed by using the single-likelihood ancestor counting (SLAC), fixed effects likelihood (FEL), internal branch FEL (iFEL), random effects likelihood (REL), mixed effects model of evolution (MEME) and fast unconstrained Bayesian approximation (FUBAR) methods implemented in the Datamonkey Web-Server. Significance level settings for these tests were considered per the default values available on the Datamonkey Web-server [35]. Amino acid property-based predictions of positive selection were determined by using the TreeSAAP program. Significant changes in amino acid properties were inferred for categories 6–8 with their relative percent probability set at 99.9% [36]. Evidences of genetic recombination were analyzed by using the Recombination Detection Program version 4 (RDP4) that includes RDP4, GENCONV, Bootscanning, the Maximum Chi Square, CHIMAERA, Sister Scanning and 3SEQ methods [37]. The fixation index (*Fst*) was deployed to evaluate the level of population differentiation due to genetic structure by using different hierarchical analyses of molecular variance implemented in the Arlequin software version 3.11 [38]. Significance levels of the fixation indices were determined by permutation test. Phylogenetic trees were constructed using the maximum likelihood method based on the best substitution model for the sequence data that showed the lowest Bayesian Information Criterion (BIC) scores [34]. The reliability of clustering patterns in the phylogenetic tree was assessed by 1,000 bootstrap pseudoreplicates. BCPRED server was used to predict B-cell epitopes with an epitope length of 20 amino acids, set to 90% classifier specificity [39]. PREDIVAC server was employed to stimulate the MHC-II binding peptide [40]. It is a cutting-edge tool to predict CD4+ T-cell epitopes because it has over 95% coverage of human HLA class II DR protein diversity. In the present study, HLA-DR alleles were restricted to DRB1*1202, DRB1*1502, DRB1*0701, DRB1*1501, and DRB5*1602 due to its predominant frequencies among Thai population [41]. The threshold PREDIVAC score was set at 70 to screen MHC-II binding antigenic peptides.

## Results

### Diversity in *PvMSP-7E* of Thai isolates

Of 110 *P*. *vivax*-infected blood samples, 92 isolates contained single genotypes of the block 6 of *PvMSP-1* and yielded non-superimposed signals of the electropherograms of the *PvMSP-7E* sequences, suggesting single clone infections. Since Yala and Narathiwat provinces are located next to each other and malaria transmission has been almost similar, *P*. *vivax* isolates from these provinces are considered to be the same population. Therefore, the *PvMSP-7E* sequences in this study were defined by geographic origins as Tak (n = 46), Ubon Ratchathani (n = 22) and Yala-Narathiwat (n = 24). However, Tak population was further subdivided into 2 populations based on collection period, i.e. 2008–2009 (n = 28) and 1996 (n = 18)(Table 1). In total, 52 haplotypes were identified among 92 Thai isolates, comprising 194 nucleotide substitutions, 185 segregating sites and 9 insertion/deletions. Haplotype #1 was most common and shared between *P*. *vivax* populations from Ubon Ratchathani (n = 2) and Yala-Narathiwat (n = 14). Likewise, haplotypes #15-#17 co-existed in Tak and Ubon Ratchathani. However, the remaining 48 haplotypes were not shared between these endemic areas (S2 Fig). Although the level of nucleotide diversity for Tak population was higher than those for Ubon Ratchathani and Yala-Narathiwat populations, the differences were not statistically significant (Z-test, *p* > 0.05). Meanwhile, the number of haplotypes and haplotype diversity for Yala-Narathiwat population were remarkably lower than those for Tak and Ubon Ratchathani populations, indicating limited number of variants and more uneven distribution of haplotypes in Yala and Narathiwat provinces.

**Table 1:** Sequence diversity in the *PvMSP-7E* gene of *P*. *vivax* populations in Thailand.

	n	M	S	Indel	H	*h* ± S.D.	π ± S.E.
Tak	46	191	183	9	34	0.986 ± 0.007	0.0620 ± 0.0046
Tak 1996	18	182	174	9	16	0.987 ± 0.023	0.0496 ± 0.0039
Tak 2008–2009	28	189	181	9	22	0.984 ± 0.013	0.0677 ± 0.0050
Yala-Narathiwat	24	117	116	9	3	0.540 ± 0.062	0.0514 ± 0.0048
Ubon Ratchathani	22	161	153	9	19	0.987 ± 0.018	0.0586 ± 0.0047
Total	92	194	185	9	52	0.958 ± 0.013	0.0613 ± 0.0047

n: number of isolates, M: number of mutations, S: number of segregating sites, Indel: number of insertions or deletions, H: number of haplotypes, *h*: haplotype diversity, π: nucleotide diversity, S.E.: standard error.

### Sequence variation in the 5’ and the 3’ regions of *PvMSP-7E*

**Fig 1:**
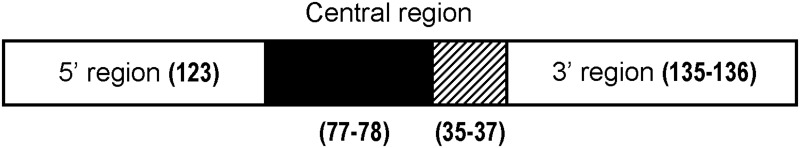
Schematic representation of *PvMSP-7E* depicting conserved (open boxes), variable trimorphic (filled black box) and dimorphic (hatched box) regions. The number of codons for each region is in parentheses.

Alignment of the complete *PvMSP-7E* sequences of isolates in this study with the Salvador 1 sequence has revealed two regions with low nucleotide diversity (π = 0.0224 and 0.0273) located in the 5’ and 3’ regions, spanning 123 and 135–136 codons, respectively (Fig 1). It is noteworthy that 51 nucleotides at the 5’ end and 15 nucleotides at the 3’ end of the gene were not analyzed among Colombian isolates; these regions contained 4 nonsynonymous codon changes at residues F12L, F14L, S17C and L368F [19]. In total 20 and 51 nucleotide substitutions were observed in the 5’ and 3’ regions, respectively. Of these, 69 nucleotide substitutions were dimorphic and 2 substitutions at positions 786 and 926 at the 3’ region were trimorphic, i.e. 3 bases are found at positions wherever substitutions occur. Most Thai isolates (94.6%) had a deletion of codon 312 coding for proline in the 3’ region, whereas 21 (22.8%) and 26 (28.3%) isolates contained TTA (leucine) and GAA (glutamine) insertions between codons 150 and 151, and between codons 215 and 216, respectively (positions after the coding region of the Salvador 1 sequence).

### Sequence variation in the central region of *PvMSP-7E*

**Fig 2:**
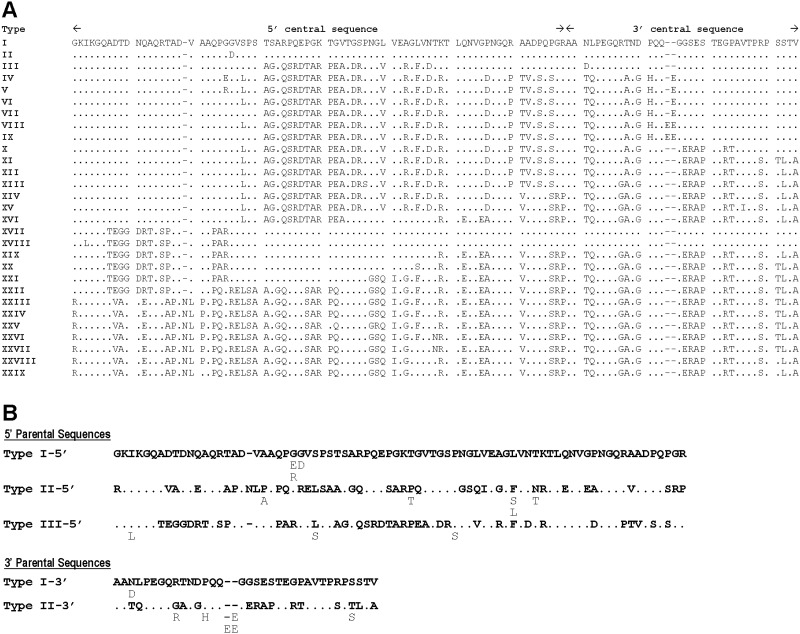
Sequence variation in the central region of PvMSP-7E. (A) Boundaries of the N- and C-terminal portions. (B) Parental alleles of the N- and C-terminal subregions. Dots and dashes are identical residues and deletion/insertion, respectively. Variant amino acids are listed beneath each parental sequence.

The nucleotide diversity of the central region of *PvMSP-7E*, encompassing codons 124–234 of the Salvador 1 strain (Fig 1), displayed an order of magnitude greater than those for the 5’ and 3’ regions and the differences were statistically meaningful (*p* < 0.0001)(Table 2). Closer looks into the central amino acid sequences have revealed 29 allelic types (Fig 2A). Repeat motifs were undetectable in this gene. Importantly, the N-terminal part of the central region spanning residues 124–200 exhibited mosaic organization of sequences that could have been generated by genetic shuffling among three parental types, represented by the Salvador 1 strain (type I-5’), the APH5 isolate from Tak province (type II-5’) and an unknown strain (type III-5’)(Fig 2A). Meanwhile, the C-terminal portion of the remaining central region could have arisen from 2 parental types (Fig 2B). Therefore, nucleotide sequences encoding these N- and C-terminal parts of the central region are referred here as 5’-trimorphic and 3’-dimorphic subregions, containing 77–78 and 35–37 codons, respectively (Fig 1). The level of nucleotide diversity for the 5’-trimorphic region was significantly greater than that for the 3’-dimorphic region (*p* < 0.005)(Table 2). Furthermore, 2 indels were found in the central region of this gene, one in the 5’-trimorphic and the other in the 3’-dimorphic subregions.

**Table 2:** Nucleotide diversity (π) and number of synonymous (*d*_S_) and nonsynonymous (*d*_N_) nucleotide substitutions per site in *PvMSP-7E* among Thai isolates.

Region	n	M	*h*	π ± S.E.	*d*_S_ ± S.E.	*d*_N_ ± S.E.
5’	123	20	13	0.0224 ± 0.0050	0.0889 ± 0.0277**	0.0090 ± 0.0038
Central	112–113	122	20	0.1598 ± 0.0150§§§§	0.1125 ± 0.0261	0.1874 ± 0.0195*
5’-trimorphic	77	99	16	0.1939 ± 0.0198##	0.1483 ± 0.0394	0.2164 ± 0.0245
3’-dimorphic	36	23	10	0.0973 ± 0.0222	0.0349 ± 0.0253	0.1300 ± 0.0304*
3’	135–136	52	42	0.0273 ± 0.0044	0.0799 ± 0.0194***	0.0109 ± 0.0033
Total	369–371	194	52	0.0614 ± 0.0047	0.0919 ± 0.0123*	0.0549 ± 0.0056

n: number of codons, M: number of mutations, *h*: number of haplotypes. Tests of the hypothesis that *d*_S_ equals *d*_N_;. Tests of the hypotheses that π in the central region equals the corresponding values in the 5’ and the 3’ regions; Tests of the hypotheses that π in the 5’ trimorphic region equals that in the 3’ dimorphic region;

* *p* < 0.05;

** *p* < 0.005;

*** *p* < 0.001

^**§§§§**^ *p* < 0.0001.

^##^ *p* < 0.005.

### Protein secondary structure prediction

**Fig 3:**
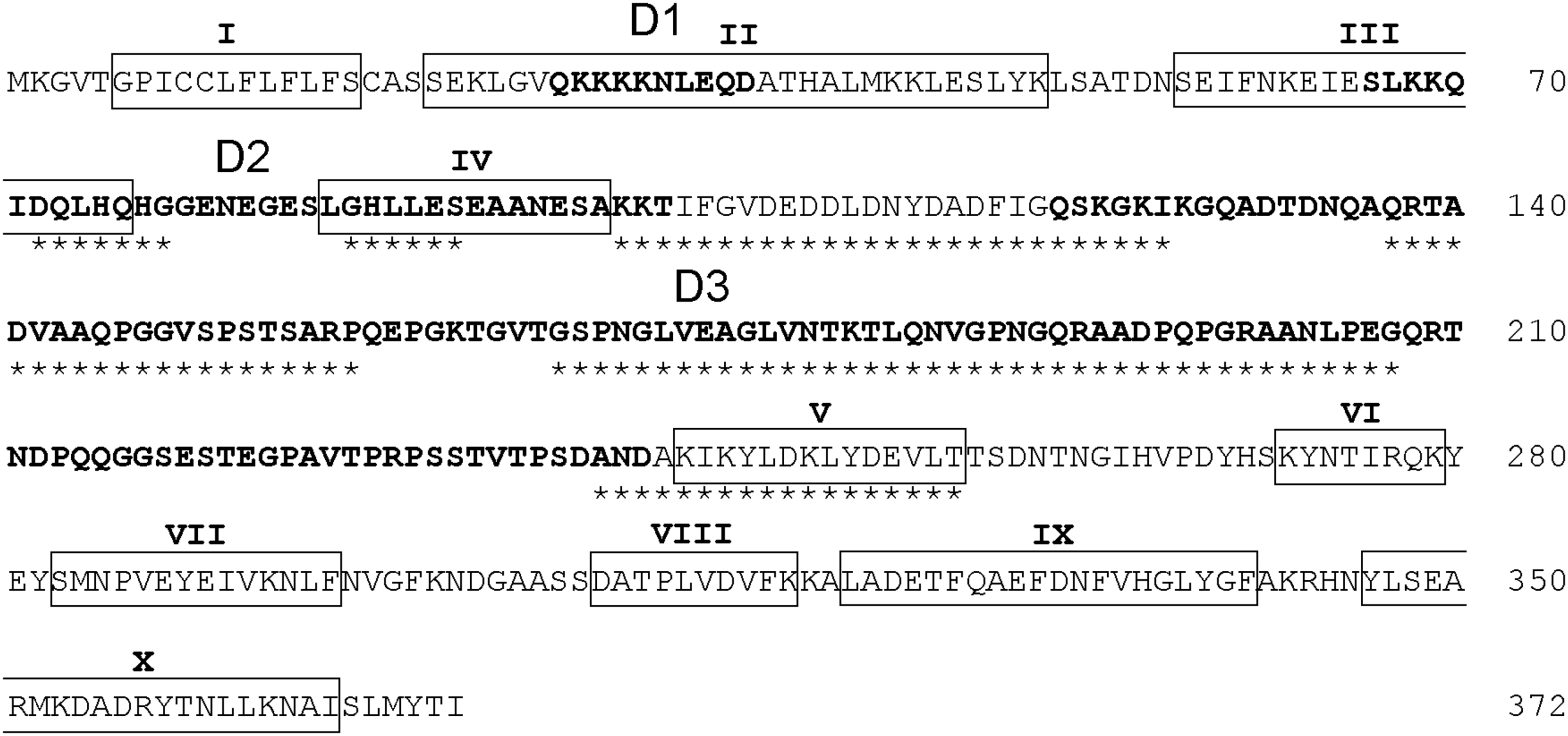
Predicted protein secondary structure of PvMSP-7E. α-helices and coiled structures are boxed and unboxed, respectively. Disordered protein regions are shown in bold. Protein-protein binding regions are marked underneath with asterisks.

Protein secondary structure prediction of PvMSP-7E by using DeepCNF method implemented in the RaptorX-Property Web-Server has revealed 10 α-helical domains. Four α-helices were located in the N-terminal and the remaining 6 α-helices at the C-terminal (Fig 3). The majority of the non-helical regions seem to contain random coil motifs. Analysis using the MetaDisorder series implemented in the GeneSilico Metadisorder service has identified 3 intrinsically unstructured or disordered regions that span codons 27–36, 46–102 and 121–241, referred here as domains D1, D2 and D3, respectively. Most of the protein-protein interaction regions were mapped in the central portion of PvMSP-7E (Fig 3).

### Selective pressure on *PvMSP-7E*

Departure from neutrality in the *PvMSP-7E* locus by comparison of *d*_S_ and *d*_N_ has shown that *d*_S_ was significantly greater than *d*_N_ in both 5’ and 3’ regions (*p* < 0.005), suggesting purifying selection in these domains. By contrast, the central region seems to be under positive selection because *d*_N_ significantly exceeded *d*_S_ (*p* < 0.05)(Table 2). Similar results were obtained when analysis was performed on sequences from each parasite population (S1 Table). Further analysis of the central region has revealed that *d*_N_ significantly outnumbered *d*_S_ in the 3’-dimorphic (*p* < 0.05), but not in the 5’-trimorphic region (*p* > 0.05) (Table 2). To explore whether selective pressure could have operated on specific regions of the protein, the rates of synonymous and nonsynonymous substitutions per site were determined for each domain according to the predicted protein secondary structure. Owing to the paucity of mutation sites in α-helical domains except α-helix-I, 3 adjacent helical regions were combined for analysis. Results revealed that *d*_S_ significantly exceeded *d*_N_ in α-helical domains II-IV, V-VII and VIII-X, suggesting purifying selection in these regions. Evidence of purifying selection was also observed in the predicted disordered domain 2 (D2). The remaining non-helical regions and the D1 domain exhibited no significant difference between *d*_S_ and *d*_N_. On the other hand, the α-helix-I domain displayed greater value of *d*_N_ than *d*_S_ and the difference was statistically significant, implying positive selection in this region. In domain D3, *d*_N_ outnumbered *d*_S_ but the difference was not significant. However, positive selection was detected in the 3’ portion of the D3 domain corresponding to the 3’-dimorphic subregion where significantly greater *d*_N_ than *d*_S_ was observed (Tables 2 and 3).

**Table 3:** Number of synonymous (*d*_S_) and nonsynonymous (*d*_N_) substitutions per site in relation to protein secondary structure prediction of PvMSP7E.

Predicted domain*	Nucleotides	*d*_S_ ± S.E.	*d*_N_ ± S.E.
α-helix I	36	0.0000 ± 0.0000	0.0608 ± 0.0310#
α-helices II-IV	192	0.0776 ± 0.0372#	0.0040 ± 0.0035
α-helices V-VII	108	0.0992 ± 0.0480#	0.0005 ± 0.0005
α-helices VIII-X	156	0.0480 ± 0.0215#	0.0051 ± 0.0029
α-helices II-X	456	0.0699 ± 0.0184##	0.0035 ± 0.0018
Remaining non-helical regions	630	0.1160 ± 0.0202	0.0967 ± 0.0101
Disorder I	30	0.0000 ± 0.0000	0.0205 ± 0.0208
Disorder II	171	0.2023 ± 0.0867#	0.0040 ± 0.0037
Disorder III	369	0.1116 ± 0.0250	0.1667 ± 0.0176

Tests of the hypothesis that *d*_S_ equals *d*_N_:

^#^ *p* < 0.05;

^##^ < 0.0001.

* Domains are demarcated as in Fig 3.

Codon-based tests for departure from neutrality using SLAC, FEL, iFEL, REL, FUBAR and MEME methods implemented in the Datamonkey Wed-Server have identified 2, 8, 11, 39, 12 and 20 positively selected sites, respectively. Likewise, the TreeSAAP program that determined significant alteration in various physicochemical properties of substituted amino acids could detect 18 positively selected sites. Meanwhile, SLAC, FEL, iFEL, REL and FUBAR identified 23, 38, 31, 30 and 24 negatively selected sites, respectively. However, these methods could potentially generate some false positive and negative results. Therefore, a consensus of concordant results for positively and negatively selected residues from at least 2 methods was considered herein and listed in Tables 4 and 5. It is noteworthy that the majority (21 of 26) of codons displaying positive selection were mapped in α-helix-I and domains predicted to be disordered protein. On the other hand, most negatively selected codons (29 of 34) were found outside these domains. The distribution of these positively and negatively selected sites in relation with α-helix and disordered regions was significant difference (*p* < 0.0001, Fischer exact probability test).

**Table 4:** Codon-based analysis of positive selection in *PvMSP-7E*.

Region	Codon	Amino acid		Test method						
		Sal-1	Variant	SLAC	FEL	IFEL	REL	FUBAR	MEME	TreeSAAP
Helix-I	12	F	L	**✓**	**✓**		**✓**	**✓**	**✓**	
Helix-I	14	F	L		**✓**	**✓**	**✓**	**✓**	**✓**	
Helix-I	17	S	C		**✓**		**✓**	**✓**	**✓**	**✓**
Helix-II/Disorder-I	29	K	R				**✓**	**✓**		
Disorder-II	83	G	E				**✓**	**✓**		
Disorder-III	131	D	V / E				**✓**			**✓**
Disorder-III	132	T	A / G		**✓**	**✓**		**✓**	**✓**	
Disorder-III	139	T	A / P			**✓**	**✓**			
Disorder-III	142	V	N				**✓**		**✓**	
Disorder-III	143	A	L				**✓**		**✓**	
Disorder-III	149	V	R						**✓**	**✓**
Disorder-III	150	S	E / L		**✓**	**✓**	**✓**	**✓**	**✓**	**✓**
Disorder-III	160	P	S / T		**✓**	**✓**	**✓**			
Disorder-III	164	G	Q / E			**✓**	**✓**			
Disorder-III	170	N	G					**✓**	**✓**	
Disorder-III	175	A	G / R		**✓**	**✓**	**✓**	**✓**	**✓**	**✓**
Disorder-III	188	P	A / D			**✓**	**✓**		**✓**	
Disorder-III	192	R	P			**✓**	**✓**			**✓**
Disorder-III	193	A	V / T			**✓**	**✓**		**✓**	**✓**
Disorder-III	203	N	T / D				**✓**		**✓**	
Disorder-III	209	R	G				**✓**			**✓**
Disorder-III	229	R	S			**✓**	**✓**		**✓**	
Non-helix	262	G	W					**✓**	**✓**	**✓**
Non-helix	263	I	M				**✓**		**✓**	
Helix-VII	285	N	K / D				**✓**	**✓**	**✓**	
Non-helix	368	L	F	**✓**	**✓**		**✓**	**✓**	**✓**	

Concordant results from 2 or more tests are shown. Tick marks indicate significant positive selection. For codon-based tests, *p* values are based on default values in the Datamonkey web-server. For TreeSAAP, *p* values < 0.001 in categories 5–8 are considered to be significant differences in amino acid properties.

**Table 5:** Codon-based analysis of negative selection in *PvMSP-7E*.

Region	Codon	Sal-1	Test method				
			SLAC	FEL	IFEL	REL	FUBAR
Helix-II	46	E	**✓**	**✓**	**✓**	**✓**	**✓**
Helix-II	48	L	**✓**	**✓**	**✓**	**✓**	**✓**
Non-helix	51	L	**✓**	**✓**	**✓**	**✓**	**✓**
Non-helix	52	S	**✓**	**✓**	**✓**	**✓**	**✓**
Non-helix	53	A	**✓**	**✓**	**✓**	**✓**	**✓**
Non-helix	56	N	**✓**	**✓**	**✓**	**✓**	**✓**
Helix-III	63	E	**✓**	**✓**	**✓**	**✓**	**✓**
Helix-III	64	I	**✓**	**✓**	**✓**	**✓**	**✓**
Helix-III	72	D	**✓**	**✓**	**✓**	**✓**	**✓**
Non-helix	78	G	**✓**	**✓**	**✓**	**✓**	**✓**
Disorder-III	127	K		**✓**	**✓**		
Disorder-III	134	N	**✓**			**✓**	
Disorder-III	148	G		**✓**	**✓**	**✓**	**✓**
Disorder-III	155	A		**✓**		**✓**	**✓**
Disorder-III	238	D		**✓**	**✓**		
Non-helix	242	A		**✓**	**✓**	**✓**	
Helix-V	247	L	**✓**	**✓**	**✓**	**✓**	**✓**
Helix-V	248	D	**✓**	**✓**	**✓**	**✓**	**✓**
Helix-V	249	K	**✓**	**✓**	**✓**	**✓**	**✓**
Helix-V	250	L		**✓**	**✓**	**✓**	
Helix-V	256	T	**✓**	**✓**	**✓**	**✓**	**✓**
Non-helix	257	T	**✓**	**✓**	**✓**	**✓**	**✓**
Non-helix	259	N	**✓**	**✓**	**✓**	**✓**	**✓**
Non-helix	261	T	**✓**	**✓**	**✓**	**✓**	**✓**
Non-helix	267	D	**✓**	**✓**	**✓**	**✓**	**✓**
Helix-VI	273	N		**✓**	**✓**		
Helix-VII	288	E	**✓**	**✓**	**✓**	**✓**	**✓**
Helix-VII	295	L	**✓**	**✓**	**✓**	**✓**	**✓**
Non-helix	307	S		**✓**	**✓**	**✓**	
Helix-VIII	313	L		**✓**		**✓**	
Helix-IX	331	D		**✓**	**✓**		
Helix-IX	335	H		**✓**	**✓**		
Helix-X	354	D	**✓**	**✓**	**✓**	**✓**	**✓**
Helix-X	365	A	**✓**	**✓**	**✓**	**✓**	**✓**

Concordant results from 2 or more tests are shown. Tick marks indicate significant *p* values based on default option in the Datamonkey Web-Server.

### Recombination

Intragenic recombination in the *PvMSP-7E* locus was evidenced by various recombination parameters. The RDP package incorporating RDP, GENCONV, Bootscan, MaxChi, Chimera, Siscan and 3Seq identified 11, 8, 8 and 1 unique events by at least one of these tests in populations from Tak collected during 2008–2009, Tak in 1996, Ubon Ratchathani and Yala-Narathiwat, respectively. More than half of the nucleotide sites (29 of 56) involved in these recombination events were located in the central region of the gene (S2 Table).

### Population differentiation

Genetic differentiation between populations was measured from the fixation index that ranges between 0 to 1 (or 100%), indicating no subdivision between populations and complete genetic isolation between populations, respectively. The *Fst* values based on *PvMSP-7E* between *P*. *vivax* populations and their *p* values are shown in Table 6. The *Fst* values were relatively high and reached highly significant levels (p < 10^−5^) between populations from Tak (all collection periods) and Yala-Narathiwat (21.75%), and between Ubon Ratchathani and Yala-Narathiwat (19.44%), implying genetic differentiation or limited gene flow between these endemic areas. Although the *Fst* value between Ubon Ratchathani and Tak (all collection period) was low (0.82%), it was significantly deviated from zero (*p* = 0.045). When Tak isolates were considered separately according to sampling years, significant deviation from zero of the *Fst* values were also observed when comparisons were performed with Yala-Narathiwat population. Likewise, genetic differentiation was observed between populations from Ubon Ratchathani and Tak collected in 1996 (*p* = 0.018) despite a small *Fst* value. However, the *Fst* value between populations from Ubon Ratchathani and Tak collected during 2008–2009 was not significant, suggesting that more gene flow between these areas occurred rather recently. Nevertheless, low level of genetic divergence between Tak populations collected in 1996 and during 2008 and 2009 was observed (*p* = 0.108), implying chronological genetic stability of Tak population.

**Table 6:** Interpopulation variance indices of *P*. *vivax* populations in Thailand inferred from *PvMSP-7E*.

**Province**	**Tak 1996**	**Tak 2008–2009**	**Tak (all)**	**Ubon Ratchathani**	**Yala-Narathiwat**	**Colombia**
Tak 1996		0.396	-	0.018	< 10^−5^	-
Tak 2008–2009	0.0263		-	0.108	< 10^−5^	-
Tak (all)	-	-		0.045	< 10^−5^	0.036
Ubon Ratchathani	0.0108	0.0075	0.0082		< 10^−5^	0.027
Yala-Narathiwat	0.2473	0.2328	0.2175	0.1944		< 10^−5^
Colombia	-	-	0.0070	0.0131	0.2387	

Pairwise *Fst* values (lower diagonal) between *P*. *vivax* populations and their *p* values by permutation test (upper diagonal). Dashes indicate no comparison was done.

### Phylogenetic analysis

**Fig 4:**
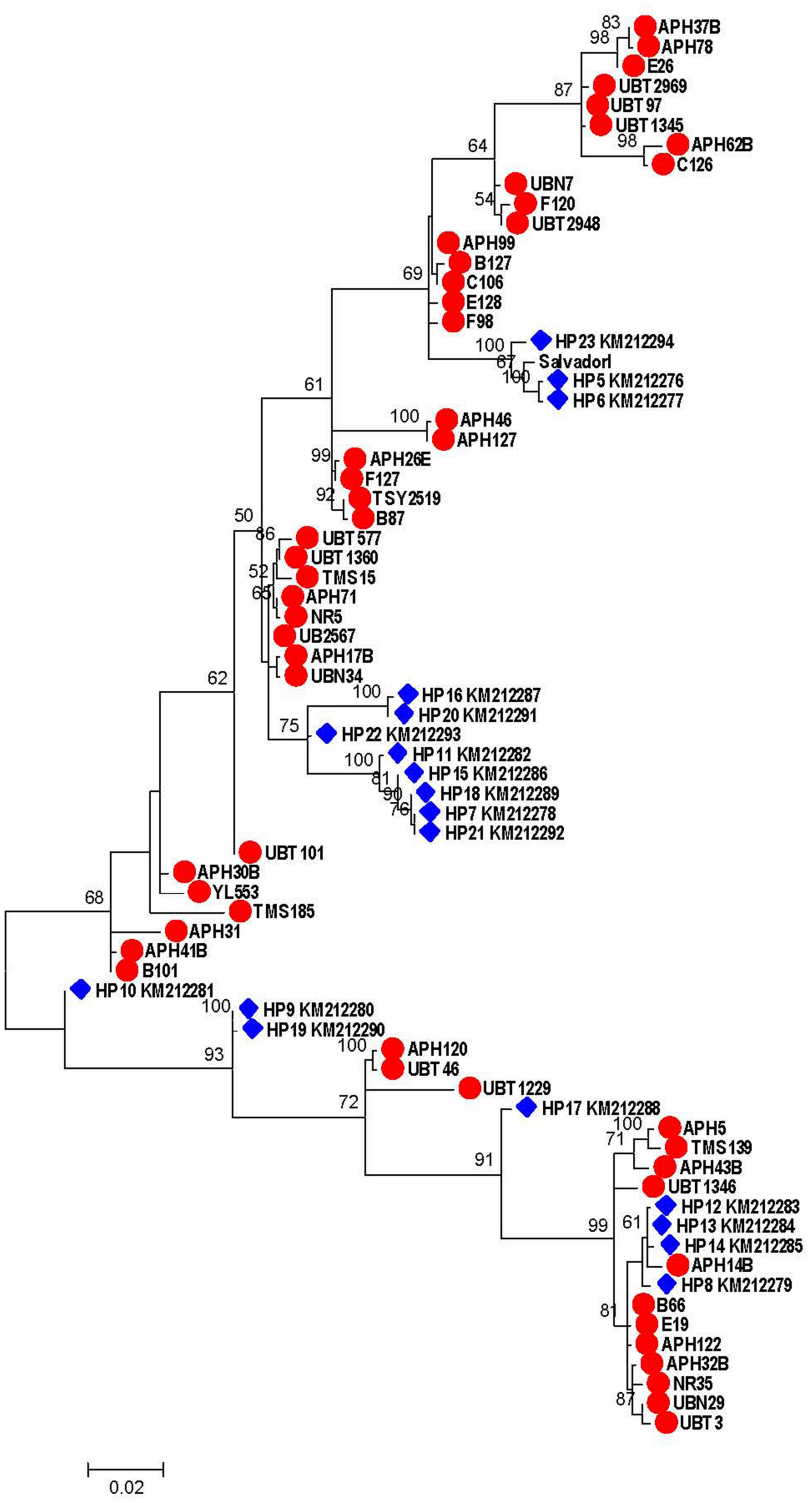
Maximum likelihood phylogenetic tree of *PvMSP-7E* based on Hasegawa-Kishino-Yano model and gamma distributed with invariant sites. Tree was constructed using distinct sequences of Thai and Colombian isolates (closed triangle) comparing with the Salvador I strain (closed circle). Bootstrap values >50% are shown.

Maximum likelihood tree was constructed using Hasegawa-Kishino-Yano model and gamma distributed with invariant sites that gave the lowest BIC score. Phylogenetic analysis of Thai isolates and those reported from Colombia has shown no geographic clustering of the *PvMSP-7E* sequences (Fig 4). Although the tree topology displayed 2 clusters of sequences, the bootstrap value was low. Mosaic organization in the central region of the gene, probably due to recurrent interallelic recombination events throughout this locus, could lead to phylogenetic homogenization of *PvMSP-7E*.

### Predicted linear B-cell and helper T-cell epitopes

Most of the predicted B-cell epitopes were identified in the central region of PvMSP-7E (S3 Fig). On the other hand, the putative CD4+ T cell epitopes for common HLA-DRB1 haplotypes in Thai population, i.e. DRB1*0701, DRB*1202, DRB1*1501, DRB1*1502 and DRB5*1602 [41], were predicted to occur in all domains of the protein. However, amino acid substitutions in these epitopes seem to alter predicted HLA-binding scores (S3 Table).

## Discussion

Primary processing of PfMSP-7 generates two protein fragments with molecular weight of 20-kDa and 33-kDa. Although the fate of the former N-terminal fragment remains to be addressed, the latter C-terminal fragment is found to be associated with the PfMSP-1 precursor protein [10]. Secondary processing of PfMSP-7 yields 19- or 22-kDa proteins, in which the cleavage sites occur after glutamine residues, i.e. between glutamine and glutamic acid, and glutamine and serine, respectively [42]. Currently it is unknown whether PvMSP-7 undergoes proteolytic processing akin to PfMSP-7. However, a consensus for *P*. *falciparum* subtilisin 1 (PfSUB1) cleavage site seems to be present in PvMSP7E (S4 Fig) [43].

Sequence analysis of the *PvMSP-7E* gene of Thai isolates has shown that both 5’ and 3’ regions displayed low levels of nucleotide diversity that are in line with previous analysis of the Colombian samples [19]. Although the 3’ region contained more nucleotide substitutions than the 5’ region, the levels of nucleotide diversity of these regions did not significantly differ (Table 2). However, evidences of purifying selection were observed in both regions, suggesting that structure or function of the protein could affect the rate of synonymous and nonsynonymous substitutions in these regions. Codon-based tests for deviation from neutrality also supported that most negatively selected codons occurred in these regions. Further analysis, taking into account the predicted protein secondary structure, has revealed evidences of purifying selection in all helical domains except α-helix-I, implying structural or functional constraints in most α-helical structure of the protein. On the other hand, α-helix-I was under positive selection because *d*_N_ was significantly greater than *d*_S_. It is noteworthy that the putative signal peptide of PvMSP-7E spanned α-helix-I domain that seems to be shed from the precursor protein without any association with the MSP-1 complex [9]. To date it is unknown whether the N-terminal signal peptide would be immunogenic during malaria infection. However, positive selection in the N-terminal signal peptide of this protein could imply its role in immune evasion process because amino acid substitutions in α-helix-I domain (residues 12, 14, 16 and 17) could alter CD4+ T helper cell epitopes’ predicted scores for peptide binding to the common HLA-DRB1 haplotypes in Thai population (S3 Table) [40, 41].

Although signals of recombination events have been detected across *PvMSP-7E*, the majority of potential recombination breakpoints seem to be more pronounced in the central region than the remainders. Therefore, a higher level of nucleotide diversity in the central domain than those in the 5’ and 3’ regions could partly stem from intragenic recombination between distinct alleles. The 5’ domain of the central region exhibits mosaic organisation of sequences that could be plausibly generated by intragenic recombination among 3 putative parental alleles. Meanwhile, the 3’ domain of the central region could be derived from recombination between dimorphic parental alleles. It is likely that intragenic recombination between distinct alleles during sexual reproduction in anopheline vector could enhance genetic diversity at this locus, implying that effective vector control could reduce genetic diversity of parasite population. Recombination has a local influence on sequence diversity, where it stabilizes adaptive traits or removes deleterious variants [44]. Intriguingly, the boundary between the conserved 5’region and the central variable region of *PvMSP-7E* seems to be around the predicted processing site as inferred from a consensus of amino acid residues observed in subtilisin 1 of *P*. *falciparum* (PfSUB1)(Fig 2 and S4 Fig) [43]. These features could be related to the binding domains of MSP-7 to the primary processing fragments of MSP1, in which the C-terminal part of PfMSP-7 is reportedly mediated this protein-protein interaction [5, 42].

The central region of PvMSP-7E was highly enriched in polar and charge amino acids with a number of glycine and proline residues whilst non-polar and non-charge residues were sparse in this region (Fig 2). Importantly, the entire central region of PvMSP-7E exhibited a consensus prediction of intrinsically unstructured or disordered protein. Although intrinsically disordered protein regions were also predicted to occur at two clusters in the N-terminal part (D 1 and D2) as a sub-segment in α-helix-II and region spanning helices III and IV, they were relatively short. It is important to note that most intrinsically disordered protein regions undergo transition to ordered or structured proteins upon functioning [45]. The predicted disordered domains 1 and 2 that overlapped α-helical domains II-IV in the N-terminal part of PvMSP-7E could change to their corresponding structure proteins upon contact with their respective, yet unknown targets. Importantly, the N-terminal part of MSP-7 has been recently demonstrated to be a ligand for the host’s P-selectin [14]. Because P-selectin is a cell adhesion molecule on the surface of activated endothelial cells and platelets that plays an important role in efficient recruitment of leucocytes to the site of tissue injury during the inflammatory process, binding of malarial MSP-7 to this specific host receptor could modulate disease severity during malaria infection [46, 47]. Purifying selection in the 5’ region of *PvMSP-7E* could support functional constraint on the N-terminal part of this protein.

The occurrence of long stretch of unstructured or disordered region in the central part of PvMSP-7E could lead to increased in intrinsic plasticity that has been considered to be an important characteristic for molecular recognition of or interaction with its protein targets. The protein-protein binding regions were also predicted to be located in the central part of this protein (Fig 3). Importantly, the C-terminal MSP-7 fragment associated with MSP-1 complex has not been directly involved in erythrocyte recognition [48, 49]. Test for departure from neutrality by comparison between *d*_S_ and *d*_N_ has shown evidence for positive selection in the central region of *PvMSP-7E*. However, signals of positive selection occurred exclusively to the 3’ portion of this region corresponding to the dimorphic central domain of the protein. Besides being a potential binding region to the primary processing fragments of MSP-1, the dimorphic domain could be responsible for immune evasion. Consistently, mice immunized with *P*. *yoelii* MSP-7 could elicit antibody response, but failed to protect mice against lethal infection with the virulent strain [50]. Although the effects of sequence diversity in PvMSP-7E on host immune responses remain to be investigated, amino acid substitutions in the this protein could remarkably alter predicted scores for HLA-bindings for CD4+ T helper cell epitopes as well as predicted scores for linear B-cell epitopes, and particularly concentrated in the central domain (S3 Fig and S3 Table).

The implementation of integrated malaria control program in Thailand has markedly reduced the annual parasite incidences of the country during the past 3 decades. However, foci of malaria transmission remain in several provinces bordering Myanmar, Cambodia and Malaysia albeit being considered hypoendemic areas [51]. Analysis of genetic diversity in the *PvMSP-7E* locus of *P*. *vivax* populations from 3 major endemic areas of Thailand has shown a remarkably lower level of haplotype diversity in Yala and Narathiwat than other endemic areas, in which only 3 haplotypes circulated in the study population whereas 19 and 34 haplotypes were sampled from Ubon Ratchathani and Tak, respectively. These findings are in agreement with previous reports on analysis of other genetic loci that encode merozoite surface protein-5, apical membrane antigen-1 and thrombospondin-related adhesive protein of *P*. *vivax* in this country [22, 23, 52, 53]. Reduced haplotype diversity in *P*. *vivax* and *P*. *falciparum* populations from Yala and Narathiwat occurred as a result of population bottlenecks in the parasites caused by control measures [22]. Although reduction in number of malaria cases in Thailand occurred across endemic areas over the past decades, cross-border migration of malaria cases was common along Thai-Myanmar and Thai-Cambodia borders but rare along Thai-Malaysia border. Therefore, population bottleneck effects can be envisaged among malaria parasites in Yala and Narathiwat provinces. The recombination breakpoints in *PvMSP-7E* of *P*. *vivax* populations surveyed ranged from 1 in Yala-Narathiwat population to 11 in Tak population collected during 2008–2009. The correlation between the number of recombination breakpoints and the levels of mean haplotype diversity was approaching a significant value (*r* = 0.941, *p* = 0.059), suggesting that intragenic recombination could have partly shaped the extent of haplotype diversity. The non-zero recombination breakpoints in *PvMSP-7E* of *P*. *vivax* isolates from Yala and Narathiwat provinces further support that bottleneck effects rather than strict clonal expansion occurred in the population.

Phylogenetic analysis neither shows specific clusters of sequences for each endemic province nor unique clades for Thai or Colombian isolates. Pairwise *Fst* estimates among *P*. *vivax* populations in this study have revealed significant genetic differentiation of *P*. *vivax* population between endemic areas. However, the fixation index between populations from Tak province collected in 1996 and during 2008–2009 exhibited low and non-significant value. This is in accord with our previous analysis on genetic diversity in the *PvTRAP* locus of parasites in Thailand that displayed spatial but not temporal variation [23]. Surprisingly, the genetic differentiation between Tak collected during 2008–2009 and Ubon Ratchathani achieved a low and non-significant *F*_*ST*_ value, suggesting gene flow or genetic admixture between these parasite populations. Recent malaria epidemic in Ubon Ratchathani province during sample collection period was mainly due to forest encroachment for illegal logging by both native people and migrants from other provinces, whereas pre-epidemic malaria cases in this province were mostly indigenous cases who acquired infections locally. Undoubtedly, population migration could shape genetic diversity of *P*. *vivax* populations in Thailand.

In conclusion, our analysis revealed extensive polymorphism in the *PvMSP-7E* locus among *P*. *vivax* populations in Thailand that could have been influenced by selective pressure and intragenic recombination. The levels of haplotype diversity displayed geographic variation in which a few haplotypes were circulating in southern parasite population as a result from bottleneck effects as previously noted [22]. Differential selective pressures were observed at this locus and seem to be related with its predicted protein secondary structure, in which helices seem to be less tolerant to molecular adaptation than unstructured or disordered domains. However, functional and biological significance of these findings requires further investigations.

## Supporting information

S1 Table Nucleotide diversity (π) and number of synonymous (*d*_S_) and nonsynonymous (*d*_N_) substitutions per site in *PvMSP-7E* among *P*. *vivax* populations in Thailand. (PDF)

S2 Table Recombination breakpoints in *PvMSP-7E* of Thai isolates. (PDF)

S3 Table Putative CD4+ T cell epitopes in PvMSP-7E of the Salvador I strain and 2 Thai isolates (APH5 and APH31) for common HLA-DRB1 and HLA-DRB5 haplotypes in Thai population. (PDF)

S1 Fig Schematic diagram of nested-PCR primers used in this study. (PDF)

S2 Fig *PvMSP-7E* haplotypes among Thai isolates. (PDF)

S3 Fig Predicted linear B cell epitopes in PvMSP-7E of the Salvador I strain and 2 Thai isolates (APH5 and APH31). (PDF)

S4 Fig Secondary processing site in PfMSP7 and predicted cleavage site in PvMSP7E (down-pointing triangles). (PDF)
