# Supplementary material for: Polymorphism in merozoite surface protein-7E of Plasmodium vivax in Thailand: Natural selection related to protein secondary structure
Source: PLoS One. 2018 May 2;13(5):e0196765. doi: 10.1371/journal.pone.0196765 (PMC5931635; doi:10.1371/journal.pone.0196765)
Supplement: S1 Table — (PDF) [file pone.0196765.s001.pdf]

S1 Table. Nucleotide diversity (π) and number of synonymous (d_S) and nonsynonymous (d_N) substitutions per site in *PvMSP-7E* among *P. vivax* populations in Thailand.

Population	Region	π ± S.E.	d_S ± S.E.	d_N ± S.E.
Tak 1996	5’	0.0181 ± 0.0051	0.0690 ± 0.0262*	0.0083 ± 0.0036
	Central	0.1222 ± 0.0115	0.0809 ± 0.0206	0.1434 ± 0.0139*
	5’-trimorphic	0.1454 ± 0.0158	0.1051 ± 0.0295	0.1664 ± 0.0213
	3’-dimorphic	0.0800 ± 0.0184	0.0405 ± 0.0245	0.1008 ± 0.0263
	3’	0.0253 ± 0.0045	0.0935 ± 0.0194***	0.0138 ± 0.0033
	All	0.0496 ± 0.0041	0.0801 ± 0.0110**	0.0451 ± 0.0044
Tak 2016	5’	0.0248 ± 0.0053	0.0935 ± 0.0286**	0.0097 ± 0.0039
	Central	0.1680 ± 0.0151	0.1201 ± 0.0276	0.1942 ± 0.0213*
	5’-trimorphic	0.2007 ± 0.0205	0.1605 ± 0.0403	0.2276 ± 0.0253
	3’-dimorphic	0.1127 ± 0.0279	0.0556 ± 0.0304	0.1363 ± 0.0313
	3’	0.0352 ± 0.0052	0.1003 ± 0.0217***	0.0131 ± 0.0036
	All	0.0677 ± 0.0048	0.1029 ± 0.0135**	0.0580 ± 0.0061
Ubon Ratchathani	5’	0.0231 ± 0.0052	0.0865 ± 0.0265**	0.0089 ± 0.0038
	Central	0.1588 ± 0.0148	0.1079 ± 0.0242	0.1874 ± 0.0202*
	5’-trimorphic	0.1963 ± 0.0208	0.1432 ± 0.0377	0.2255 ± 0.0254
	3’-dimorphic	0.0910 ± 0.0182	0.0485 ± 0.0286	0.1176 ± 0.0247
	3’	0.0189 ± 0.0038	0.0622 ± 0.0165**	0.0092 ± 0.0030
	All	0.0586 ± 0.0054	0.0838 ± 0.0132*	0.0546 ± 0.0055
Yala-Narathiwat	5’	0.0198 ± 0.0051	0.1239 ± 0.0386**	0.0114 ± 0.0049
	Central	0.1449 ± 0.0170	0.1554 ± 0.0376	0.2076 ± 0.0292
	5’-trimorphic	0.1794 ± 0.0263	0.2136 ± 0.0676	0.2594 ± 0.0460
	3’-dimorphic	0.0832 ± 0.0247	0.0708 ± 0.0430	0.1241 ± 0.0356
	3’	0.0148 ± 0.0038	0.0895 ± 0.0269**	0.0106 ± 0.0054
	All	0.0514 ± 0.0051	0.1204 ± 0.0175**	0.0611 ± 0.0079

Tests of the hypothesis that d_S equals d_N: * $p < 0.05$; ** $p < 0.005$; *** $p < 0.001$.
