# Supplementary material for: Polymorphism in merozoite surface protein-7E of Plasmodium vivax in Thailand: Natural selection related to protein secondary structure
Source: PLoS One. 2018 May 2;13(5):e0196765. doi: 10.1371/journal.pone.0196765 (PMC5931635; doi:10.1371/journal.pone.0196765)
Supplement: S2 Table — (PDF) [file pone.0196765.s002.pdf]

S2 Table. Recombination breakpoints in *PvMSP-7E* of Thai isolates.

Population	Recombination breakpoints					Method (p value)						
	Total	Between positions		Between domains		RDP	GENECONV	Bootscan	Maxchi	Chimaera	SiScan	3SEQ
Tak 1996	8	9	855	5’	3’	NS	NS	NS	NS	$2.00x10^{-2}$	NS	$2.00x10^{-6}$
		10	561	5’	Central	$4.90x10^{-13}$	$4.80x10^{-10}$	$2.00x10^{-12}$	$5.00x10^{-12}$	$3.23x10^{-12}$	$7.75x10^{-24}$	$4.94x10^{-24}$
		164	561	5’	Central	$1.52x10^{-11}$	$3.81x10^{-10}$	$5.33x10^{-8}$	$2.35x10^{-15}$	$1.17x10^{-13}$	$5.01x10^{-18}$	$4.90x10^{-26}$
		302	728	5’	3’	NS	$3.77x10^{-7}$	$4.11x10^{-9}$	$3.41x10^{-7}$	$3.23x10^{-5}$	$7.03x10^{-4}$	$1.82x10^{-11}$
		302	855	Central	Central	$1.11x10^{-8}$	$5.50x10^{-10}$	$8.74x10^{-13}$	$3.34x10^{-9}$	$3.23x10^{-8}$	$9.31x10^{-4}$	$2.42x10^{-9}$
		416	957	Central	3’	$1.24x10^{-2}$	$1.65x10^{-3}$	NS	$3.04x10^{-4}$	$4.98x10^{-6}$	$7.13x10^{-16}$	$1.28x10^{-8}$
		447	558	Central	Central	NS	NS	NS	NS	NS	NS	$5.77x10^{-8}$
		562	612	Central	Central	NS	$3.89x10^{-3}$	NS	NS	NS	NS	$1.15x10^{-5}$
Tak 2011-2016	11	33	302	5’	5’	NS	NS	NS	NS	NS	NS	$6.85x10^{-3}$
		164	603	5’	Central	$1.24x10^{-13}$	$2.54x10^{-12}$	$2.39x10^{-16}$	$1.12x10^{-14}$	$7.38x10^{-15}$	$2.62x10^{-11}$	$1.48x10^{-25}$
		299	569	5’	Central	$1.59x10^{-8}$	$4.55x10^{-5}$	$1.61x10^{-8}$	$1.35x10^{-12}$	$1.08x10^{-10}$	$2.29x10^{-13}$	$2.94x10^{-20}$
		302	533	5’	Central	NS	$5.57x10^{-10}$	$1.09x10^{-12}$	$9.32x10^{-12}$	$9.32x10^{-12}$	$7.41x10^{-23}$	$3.77x10^{-26}$
		446	560	Central	Central	NS	NS	$7.29x10^{-3}$	NS	NS	NS	$1.02x10^{-7}$
		459	520	Central	Central	NS	NS	NS	NS	NS	$9.53x10^{-11}$	NS
		561	1099	Central	3’	$6.71x10^{-10}$	$4.47x10^{-9}$	$1.86x10^{-10}$	$8.69x10^{-14}$	$3.44x10^{-13}$	$6.34x10^{-12}$	$2.23x10^{-25}$
		645	726	Central	3’	$1.77x10^{-3}$	$7.61x10^{-3}$	$1.80x10^{-3}$	$4.09x10^{-3}$	$2.83x10^{-3}$	$6.91X10^{-7}$	$9.60x10^{-6}$
		687	834	Central	3’	NS	NS	NS	NS	NS	NS	$6.85x10^{-3}$
		727	884	3’	3’	NS	$2.19x10^{-3}$	$3.56x10^{-4}$	NS	NS	$9.07x10^{-3}$	$4.74x10^{-6}$
		888	1038	3’	3’	NS	$6.53x10^{-5}$	$1.38x10^{-3}$	$3.27x10^{-6}$	$1.15x10^{-5}$	$5.52x10^{-9}$	$6.91x10^{-11}$
Ubon Ratchathani	8	14	536	5’	Central	NS	NS	NS	$1.74x10^{-2}$	$8.43x10^{-3}$	$5.18x10^{-10}$	$6.20x10^{-8}$
		68	784	5’	3’	NS	NS	NS	NS	NS	NS	$7.92x10^{-4}$
		302	536	5’	Central	$5.80x10^{-7}$	$4.98x10^{-7}$	$7.89x10^{-11}$	$1.90x10^{-11}$	$6.19x10^{-14}$	$1.86x10^{-25}$	$2.27x10^{-23}$
		302	963	5’	3’	NS	$1.48x10^{-3}$	$5.62x10^{-5}$	$4.12x10^{-4}$	$1.93x10^{-4}$	$5.37x10^{-3}$	$1.24x10^{-9}$
		446	644	Central	Central	NS	NS	NS	NS	NS	NS	$1.53x10^{-3}$
		446	703	Central	Central	$2.14x10^{-9}$	$1.91x10^{-6}$	$2.17x10^{-9}$	$2.09x10^{-10}$	$6.21x10^{-7}$	$1.60x10^{-13}$	$6.96x10^{-14}$
		462	538	Central	Central	NS	NS	NS	NS	NS	$3.26x10^{-3}$	NS
		502	1078	Central	3’	NS	$3.10x10^{-7}$	$1.03x10^{-9}$	$3.58x10^{-11}$	$6.09x10^{-11}$	$1.81x10^{-12}$	$1.21x10^{-24}$
Yala-Narathiwat	1	628	784	Central	3’	$1.59x10^{-7}$	$9.49x10^{-9}$	$1.38x10^{-7}$	$6.04x10^{-4}$	$2.14x10^{-4}$	NS	$3.62x10^{-9}$

NS indicates not significance ($p > 0.05$).
