# Supplementary material for: Polymorphism in merozoite surface protein-7E of Plasmodium vivax in Thailand: Natural selection related to protein secondary structure
Source: PLoS One. 2018 May 2;13(5):e0196765. doi: 10.1371/journal.pone.0196765 (PMC5931635; doi:10.1371/journal.pone.0196765)
Supplement: S3 Table — (PDF) [file pone.0196765.s003.pdf]

S3 Table. Putative CD4+ T cell epitopes in PvMSP-7E of the Salvador I strain and 2 Thai isolates (APH5 and APH31) for common HLA-DRB1 and HLA–DRB5 haplotypes in Thai population.

HLA	Sequence	Residues	Domain	Score	Haplotype		
					Sal 1	APH5	APH31
DRB1*1202	FIGQSK**R**KI	118-127	N-terminal	92.42		+	
	FIGQSK**G**KI	118-127	N-terminal	85.11	+		+
	**VA**DN**E**AQR**A**	131-140	Central	85.42		+	
	**DT**DN**Q**AQR**T**	131-140	Central	<70	+		+
	V**GP**NGQR**A**A	186-195	Central	82.59	+		
	V**GD**NGQR**V**A	186-195	Central	82.59			+
	V**EA**NGQR**V**A	186-195	Central	82.59		+	
	YGFAKRHNY	338-347	C-terminal	88.22	+	+	+
	YTNLLKNAI	358-367	C-terminal	86.66	+	+	+
DRB1*1502	**F**L**F**L**FS**CAS	12-21	N-terminal	86.32	+		+
	**L**L**L**L**FC**CAS	12-21	N-terminal	<70		+	
	LDNYDADFI	111-120	N-terminal	80.92	+	+	+
	E**G**G**F**V**NNR**T	175-184	Central	80.62		+	
	E**A**G**L**V**NTK**T	175-184	Central	<70	+		
	E**R**G**F**V**DTR**T	175-184	Central	<70			+
	YEIVKNLFN	289-298	C-terminal	85.01	+	+	+
	FQAEFDNFV	326-335	C-terminal	84.40	+	+	+
DRB1*0701	**F**L**F**LF**S**CAS	12-21	N-terminal	84.30	+		+
	**L**L**L**LF**C**CAS	12-21	N-terminal	<70		+	
	F**S**CASSEKL	16-25	N-terminal	87.45	+		+
	F**C**CASSEKL	16-25	N-terminal	87.45		+	
	YKLSATDNS	49-58	N-terminal	81.44	+	+	+
	LSATDNSEI	51-60	N-terminal	84.99	+	+	+
	**V**T**GS**P**NGLV**	165-174	Central	81.40	+		
	**V**T**GS**P**GSQI**	165-174	Central	79.69		+	
	**A**T**DR**P**NGVV**	165-174	Central	<70			+
	LTATPSDAN	233-242	C-terminal	80.13		+	+
	STVTPSDAN	232-241	C-terminal	<70	+		
	YEYSM**N**PVE	280-289	C-terminal	82.32	+		+
	YEYSM**K**PVE	280-289	C-terminal	<70		+	
	FKKALADET	317-326	C-terminal	84.51	+	+	+
DRB1*1501	MKGVTGPIC	1-10	N-terminal	81.77	+	+	+

HLA	Sequence	Residues	Domain	Score	Haplotype		
					Sal 1	APH5	APH31
	**F**L**F**LF**S**CAS	12-21	N-terminal	85.77	+		+
	**L**L**L**LF**C**CAS	12-21	N-terminal	<70		+	
	LFLFSCASS	13-22	N-terminal	82.31	+		+
	LLLFCCASS	13-22	N-terminal	82.31		+	
	LF**S**CASSEK	15-24	N-terminal	80.00	+		+
	LF**C**CASSEK	15-24	N-terminal	80.00		+	
	LESEAANES	90-99	N-terminal	80.85	+	+	+
	LDNYDADFI	111-120	N-terminal	86.10	+	+	+
	E**G**G**F**V**NNR**T	175-184	Central	84.19		+	
	E**A**G**L**V**NTK**T	175-184	Central	<70	+		
	E**R**G**F**V**DTR**T	175-184	Central	<70			+
	VDVFKKALA	314-323	C-terminal	81.79	+	+	+
	FQAEFDNFV	326-335	C-terminal	83.71	+	+	+
	LDKLYDEVL	247-256	C-terminal	81.62	+	+	+
	VKNLFNVGF	292-301	C-terminal	81.12	+	+	+
DRB5*1602	E**G**G**F**V**NNR**T	175-184	Central	81.03		+	
	E**A**G**L**V**NTK**T	175-184	Central	<70	+		
	E**R**G**F**V**DTR**T	175-184	Central	<70			+

Note: Prediction based on PREDIVAC: CD4+ T cell epitope prediction web-server.
