# Supplementary material for: Polymorphism in merozoite surface protein-7E of Plasmodium vivax in Thailand: Natural selection related to protein secondary structure
Source: PLoS One. 2018 May 2;13(5):e0196765. doi: 10.1371/journal.pone.0196765 (PMC5931635; doi:10.1371/journal.pone.0196765)
Supplement: S1 Fig — (PDF) [file pone.0196765.s004.pdf]

S1 Fig. Schematic diagram of nested-PCR primers used in this study.

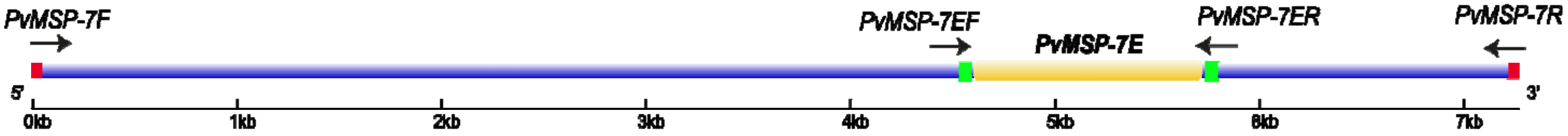

Note: The coding region of *PvMSP-7E* located on chromosome 12 of *P. vivax* was amplified by nested PCR using outer primers (red); PvMSP7F and PvMSP-7R, and inner primers (light green); PvMSP-7EF and PvMSP-7ER.
