# Supplementary material for: Polymorphism in merozoite surface protein-7E of Plasmodium vivax in Thailand: Natural selection related to protein secondary structure
Source: PLoS One. 2018 May 2;13(5):e0196765. doi: 10.1371/journal.pone.0196765 (PMC5931635; doi:10.1371/journal.pone.0196765)
Supplement: S2 Fig — (PDF) [file pone.0196765.s005.pdf]

S2 Fig. *PvMSP-7E* haplotypes among Thai isolates.

```
                    111 1111111111 1111111111 1111111111 1111111111 1111111111 1112222222 2222222222 2222222223 3333333333
             1112389223 3333333344 4444444555 5555556666 6666677777 7788888899 9990000111 1122222233 3366666880 0001112557
             2479930460 1234568902 3456789023 4567890123 4568901234 6801258934 5790145013 4901256813 4602456074 5780137670
#Salvador I  FFSKHGLGIA DTDNQARTAV AAQPGGVSPS TSARPQEPGK TGVGSPNGLV ALNTKQGPRA APPGRNLRTD PGSESGPVRS SVSNNGIQNN DAASDTTDAL
#1           LLCR.E.R.. VA..E..APN LPA.Q.RESA A.GQ...SAR PQ....GSQI GF.NREEA.V ..SRPTQGAG .ERAPRT.ST LA...W..KK EDTAAS....
#2           LLCR.EIR.. VA..E..APN LPA.Q.RESA A.GQ...SAR PQ....GSQI G..NREEA.V ..SRPTQGAG .ERAPRT.ST LA.SK....K EDTAAS....
#3           .......... .......... .......L.. AG.QSRDTAR PEADR...V. RFD.R..DPT VSS..TQ.AG H......... .....W...K EDTAAS...F
#4           .......... .......... .......... AG.QSRDTAR PEADR...V. RFD....... .....D.... .......... .....W...K EDTAAS....
#5           .......... .......... .......L.. AG.QSRDTAR PEADR...V. RFD.R..DPT VSS..TQ.AG .ERAPRT.ST LAT.SW...K EDTAAS...F
#6           ...R.E.... .......... .......L.. AG.QSRDTAR PEADR...V. RFD.R..D.V ..SRPTQGAG .ERAPRT.S. LAT.SW...K EDTAAS....
#7           LLCR.E.R.. VA..E..APN LPA.Q.RESA A.GQ...SAR PQ....GSQI G...REEA.V ..SRPTQGAG .ERAPRT.S. LA.SK....K EDTAAS...F
#8           LLCRNE...T EGGDRTSP.. ..PAR..... .......... .......... .......... .......... .......... ..T.SW...K EDTAAS.G..
#9           .......... .......... .......L.. AG.QSRDTAR PEADR...V. RFD.R..D.V ..SRPTQGAG .ERAPRTIS. LAT.SW...K EDTAAS....
#10          LLCR.E.R.. VA..E..APN L.A.Q.RESA A.GQ...SAR PQ....GSQI G...REEA.V ..SRPTQGAG .ERAPRT.S. LA...W...K EDTAAS....
#11          ..C....... .......... .......... AG.QSRDTAR PEADR...V. RFD....... .....D.... .......... .....W.... ......K...
#12          ........LT EGGDRTSP.. ..PAR..... .......... .......... .......... .......... .......... .....W...K EDTAAS.G..
#13          .......... .......... ....E..L.. AG.QSRDTAR PEADR...V. RFD.R..DPT VSS..TQ.AG H......... .........K EDTAAS....
#14          LLCRNE...T EGGDRTSP.. ..PAR..... .......... .......... .......... .......... .......... ..T.SW...K EDTAAS....
#15          .......... .......... .......... .......... .......... .......... .......... .......... .....W..DK EDTAAS....
#16          LLCR.E...T EGGDRTSP.. ..PAR..... .......... .......... ....REEA.V ..SRPTQGAG .ERAPRT.S. LA.SK....K EDTAAS...F
#17          LLCR.E.R.. VA..E..APN LPA.Q.RESA A.GQ...SAR PQ....GSQI G...REEA.V ..SRPTQGAG .ERAPRT.S. LA.SK....K EDTAAS....
#18          ..C....... .......... .......... AG.QSRDTAR PEADR...V. RFD....... .....D.... .......... .....W.... ......K...
#19          .......... .......... .......L.. AG.QSRDTAR PEADR...V. RFD.R..DPT VSS..TQ.AG H......... .....W...K EDTAAS..T.
#20          ...R.E.R.. VA..E..APN LPA.Q.RESA A.GQ...SAR PQ....GSQI GF.NREEA.V ..SRPTQGAG .ERAPRT.ST LA...W...K EDTAAS....
#21          LLCR...... .......... .......L.. AG.QSRDTAR PEADRS..V. RFD.R..DPT VSS..TQGAG .ERAPRT.S. LA.SK....K EDTAAS....
#22          .......... .......... .......... AG.QSRDTAR PEADR...V. RFD....... .....D.... .......... .....W...K EDTAAS....
#23          LLCR.E.R.. VA..E..APN LPA.Q.RESA A.GQ...SAR PQ....GSQI G...REEA.V ..SRPTQGAG .ERAPRT.S. LA.SK....K EDTAAS....
#24          .......... .......... .......... AG.QSRDTAR PEADR...V. RFD....... .....D.... .......... .....W...K EDTAAS....
#25          .......... .......... .......L.. AG.QSRDTAR PEADR...V. RFD.R..D.V ..SRPTQGAG .ERAPRT.S. LAT.SW...K EDTAAS....
#26          .......... .......... .......... .......... .......... .......... .......... .......... ......M.DK EDTAAS....
#27          .......... .......... .......... .......... .......... .......... .......... .......... .....W..DK EDTAAS....
#28          .........T EGGDRTSP.. ..PAR..... .......... .......... .......... .......... .......... ........DK EDTAAS.G..
#29          LLCR.E.R.. VA..E..APN LPA.Q.RESA A.GQ...SAR PQ....GSQI G...REEA.V ..SRPTQGAG .ERAPRT.S. LA.SK....K EDTAAS.G..
#30          LLCR.E...T EGGDRTSP.. ..PAR..... .......... .......... .......... .......... .......... ..T.SW...K EDTAAS.G..
#31          .......... .......... .......... .......... .......... .......... .......... .......... .....W...K EDTAAS....
#32          .......... .......... .....D.... .......... .......... .......... .......... .......... .....W...K EDTAAS....
#33          LLCR.E.... .......... .......... .......... .......... .......... .......... .......... ......M..K EDTAAS....
#34          .......... .......... .......... AG.QSRDTAR PEADR...V. RFD....... .....D.... .......... .....W...K EDTAAS....
#35          .......... .......... ....E..L.. AG.QSRDTAR PEADR...V. RFD.R..DPT VSS..TQ.AG H......... .........K EDTAAS....
#36          ...R.E.R.. VA..E..APN LPA.Q.RESA A.GQ...SAR PQ....GSQI G...REEA.V ..SRPTQGAG .ERAPRT.S. LA.SK....K EDTAAS....
#37          LLCR...... .......... .......L.. AG.QSRDTAR PEADR...V. RFD.R..DPT VSS..TQ.AG .ERAPRT.S. LAT.SW...K EDTAAS....
#38          .......... .......... .......L.. AG.QSRDTAR PEADR...V. RFD.R..DPT VSS..TQ.AG H......... .........K EDTAAS....
#39          LLCR.E.... .......... .......... .......... .......... .......... .......... .......... .....W..DK EDTAAS....
#40          .LCR.E.R.. VA..E..APN LPA.Q.RESA A.GQ...SAR PQ....GSQI G...REEA.V ..SRPTQGAG .ERAPRT.S. LA.SK....K EDTAAS....
#41          .......... .......... ....R..L.. AG.QSRDTAR PEADR...V. RFD.R..DPT VSS..TQ.AG H......... .........K EDTAAS...F
#42          ...R.E.R.. VA..E..APN LPA.Q.RESA A.GQ...SAR PQ....GSQI G...REEA.V ..SRPTQGAG .ERAPRT.S. LA.SK....K EDTAAS....
#43          LLCR.E...T EGGDRTSP.. ..PAR..... .......... .......... ....REEA.V ..SRPTQGAG .ERAPRT.S. LA.SK....K EDTAGS....
#44          LLCR.E...T EGGDRTSP.. ..PAR..... .......... .......... .......... .......... .......... .........K EDTAAS.G..
#45          .......... .......... .......L.. AG.QSRDTAR PEADR...V. RFD.R..DPT VSS..TQ.AG .ERAPRT... .....W..DK EDTAAS....
#46          LL........ .......... .......L.. AG.QSRDTAR PEADR...V. RFD.R..DPT VSS..TQ.AG H......... .....W...K EDTAAS....
#47          LLCR.E...T EGGDRTSP.. ..PAR..... .......... ......GSQI GF..REEA.V ..SRPTQGAG .ERAPRT.ST LAT.SW.R.K EDTAAS.G..
#48          .LCR.E...T EGGDRTSP.. ..PAR..... .......... .......... .......... .......... .......... .........K EDTAAS.G..
#49          LLCR.E.R.. VA..E..APN LPA.Q.RESA A.GQ...SAR PQ....GSQI G...REEA.V ..SRPTQGAG .ERAPRT.S. LAT.SW...K EDTAAS...F
#50          .......... .......... .......L.. AG.QSRDTAR PEADR...V. RFD.R..DPT VSS..TQ.AG H......... .....W...K EDTAAS....
#51          LLCR.E.... .......... .......... .......... .......... .......... .......... .......... ......M.DK EDTAAS....
#52          LLCR.E...T EGGDRTSP.. ..PAR..... .......... .......... .......... .......... .......... .........K EDTAAS.G..
```

Note: The distribution of haoplotypes among endemic areas are as follows: haplotype #1 (14 Yala-Narathiwat isolates and 2 Ubon Ratchathani isolates); haplotypes #2 (9 Yala-Narathiwat isolates); haplotypes #3 - #4(3 Tak isolates each); haplotypes #5 - #12 (2 Tak isolates each); haplotypes #13 - #14 (2 Ubon Ratchathani isolates each); #15 - #17 (1 Tak and 1 Ubon Ratchathani isolates each); the remaining haplotypes are found as single isolates from Tak (n=21), Ubon Ratchathani (n=13) and Yala-Narathiwat (n=1).
