# Supplementary material for: Polymorphism in merozoite surface protein-7E of Plasmodium vivax in Thailand: Natural selection related to protein secondary structure
Source: PLoS One. 2018 May 2;13(5):e0196765. doi: 10.1371/journal.pone.0196765 (PMC5931635; doi:10.1371/journal.pone.0196765)
Supplement: S3 Fig — (PDF) [file pone.0196765.s006.pdf]

S3 Fig. Predicted linear B cell epitopes in PvMSP-7E of the Salvador I strain and 2 Thai isolates (APH5 and APH31).

Salvador I	MKGVTGPICCLFLFLFSCAS**SEKLGVQKKKKNLEQDATHA**LMKKLESLYKLSATDNSEIFNKEIESLKKQIDQLHQHGGE	[80]
APH5	MKGVTGPICCLLLLLFCCASSEKLGVQKRKKNLEQDATHALMKKLESLYKLSATDNSEIFNKEIE**SLKKQIDQLHQHGGE**	[80]
APH31	MKGVTGPICCLFLFLFSCASSEKLGVQKRKKNLEQDATHALMKKLESLYKLSATDNSEIFNKEIE**SLKKQIDQLHQHGGE**	[80]

<- Central domain

Salvador I	NEGESLGHLLESEAANESAKKTIFGVDEDDLDNYDADF**IGQSKGKIKGQADTDNQAQR**TADV**AAQPGGVS-PSTSARPQE**	[159]
APH5	**NEEES**LGHLLESEAANESAKKTIFGVDEDDLDNYDADFIGQSKRKIKGQA**VADNEAQRAPDNLPAPQGRE**LS**AASGQPQE**	[160]
APH31	**NEEES**LGHLLESEAANESAKKTIFGVDEDDLDNYDADF**IGQSKGKIKGQADTDNQAQR**TADVAAQPGGVL-PSAGAQS**RD**	[159]

Central domain ->

Salvador I	**PGK**TGVTGSPNGLVEAGLVNTKTL**QNVGPNGQRAADPQPGRAAN**LPEGQRT**NDPQQGGSESTEGPAVTPRP**SSTVTPSDA	[239]
APH5	**SARPQVTGSPGS**QIEGGF**VNNRTLENVEANGQRVADPQ**SRPA**ATQPEGQGANGPQQGERAPT**ER**TAVTPSPTLTATPSDA**	[240]
APH31	**TARPEATDRPNGVVERGF**VDTRTLQNVGDNGQRVADPQSRPA**ATQPEGQGANGPQQGERAPT**ER**TAVTPSPSLTATPSDA**	[239]
Salvador I	NDAKIKYLDKLY**DEVLTTSDNTNGIHVPDYHS**KY**NTIRQKYEYSMNPVEYEIVK**NL**FNVGFKNDGAASSDATPLVD**VFKK	[319]
APH5	**NDAK**IKYLDKLY**DEVLTTSDNTNWIHVPDYHS**KY**NTIRQKYEYSMKPVEYEIVK**NLFNVGFKKEGDTSAAAS-LVDVFKK	[319]
APH31	**NDAK**IKYLDKLY**DEVLTTTDNTSWIHVPDYHS**KY**NTIRQKYEYSMNPVEYEIVK**NLFNVGFKKEGDTSAAAS-LVDVFKK	[318]
Salvador I	ALADETFQAEFDNFVHGLYGFAKRHNYLSEARMKDADRYTNLLKNAISLMYTI	[372]
APH5	ALADETFQAEFDNFVHGLYGFAKRHNYLSEARMKDADRYTNLLKNAISLMYTI	[372]
APH31	ALADETFQAEFDNFVHGLYGFAKRHNYLSEARMKDADRYTNLLKNAISLMYTI	[371]

Note: Prediction was performed using BCPred method with 90% classifier specificity implemented in the BCPREDS: B-cell epitope prediction server [39].
