# Supplementary material for: Polymorphism in merozoite surface protein-7E of Plasmodium vivax in Thailand: Natural selection related to protein secondary structure
Source: PLoS One. 2018 May 2;13(5):e0196765. doi: 10.1371/journal.pone.0196765 (PMC5931635; doi:10.1371/journal.pone.0196765)
Supplement: S4 Fig — (PDF) [file pone.0196765.s007.pdf]

S4 Fig. Secondary processing site in PfMSP7 and predicted cleavage site in PvMSP7E (down-pointing triangles).

PfMSP7 (AF390150)	PLFQNLGLFGKNVLSKVKAQ▼SETDTQSKNEQEISTQGQEV	(176/177)
PvMSP7E (PVX_082665)	DLDNYDADFIGQSKGKIKGQ▼ADTDNQAQRTADVAAQPGGV	(129/130)
Variant 1	DLDNYDADFIGQSKRKIKGQ▼AVADNEAQRAPDNLPAPQGR	
Variant 2	DLDNYDADFIGQSKGKIKGQ▼TEGGDRTQSPADVAAPARGV	

Note: Secondary processing site in PfMSP7 has been documented [10, 43]. Amino acid residues flanking known PfSUB1 cleavage sites as described by Simon de Monerri et al [43] are underlined. Amino acid residues between the cleavage sites are shown in parentheses after the sequences.
